# Health consequences for mother and baby of substantial pre-conception weight loss in obese women: study protocol for a randomized controlled trial

**DOI:** 10.1186/s13063-018-2615-6

**Published:** 2018-04-24

**Authors:** Sarah Price, Alison Nankervis, Michael Permezel, Luke Prendergast, Priya Sumithran, Joseph Proietto

**Affiliations:** 1 Department of Medicine, University of Melbourne, Heidelberg Repatriation Hospital, Waterdale Rd., Heidelberg, VIC 3081 Australia; 2 Diabetes Service, University of Melbourne, Royal Women’s Hospital, Flemington Rd., Parkville, VIC 3050 Australia; 3 Department of Obstetrics and Gynaecology, University of Melbourne, Mercy Hospital for Women, Studley Rd.,, Heidelberg, VIC 3050 Australia; 4 Department of Mathematics and Statistics, LaTrobe University, Kingsbury Drive, Bundoora, VIC 3081 Australia; 5 Department of Medicine, University of Melbourne, Royal Melbourne Hospital, Grattan St.,, Parkville, VIC 3083 Australia

**Keywords:** Pregnancy, Pre-conception, Obesity, Weight loss, Glucose metabolism, Pregnancy outcomes, Randomized trial

## Abstract

**Background:**

Current guidelines for the management of obesity in women planning pregnancy suggest lifestyle modification before conception. However, there is little evidence that lifestyle modification alters pregnancy outcomes. Bariatric surgery results in significant weight loss. This appears to reduce the risk of adverse pregnancy outcomes for the mother but may increase the risk of adverse outcomes for the infant. In order to reduce the risks of obesity-related adverse pregnancy outcomes for both mother and offspring, alternative approaches to the management of obesity in women planning pregnancy are needed.

**Methods/design:**

This study, a two-arm, parallel group, randomized control trial, will be conducted at the Metabolic Disorders Centre, University of Melbourne. This trial will recruit 164 women aged 18–38 years with a body mass index of 30–55 kg/m^2^ who plan to conceive in the next 6–12 months. Women will be randomized to one of two 12-week interventions (Group A and Group B). Group A will aim for modest weight loss (MWL; ≤ 3% body weight) using a hypocaloric diet. Group B will aim for substantial weight loss (SWL; 10–15% body weight) using a modified very low energy diet (VLED) program. All participants will be asked to comply with National Health and Medical Research Council (NHMRC) guidelines for exercise and will be provided with standard pre-pregnancy advice according to Royal Australian and New Zealand College of Obstetrics and Gynaecology guidelines. All participants will then be observed for the subsequent 12 months. If pregnancy occurs within the 12-month follow-up period, data on weight and metabolic status of the mother, and pregnancy outcomes of mother and offspring will be recorded.

The primary outcome is maternal fasting plasma glucose at 26–28 weeks’ gestation, given that this is known to correlate with pregnancy outcomes. Time to conception, live birth rate, gestational weight gain, and a composite of adverse pregnancy outcomes for mother and baby will comprise the secondary outcomes.

**Discussion:**

There is increasing emphasis on obese women losing weight before conception. To date, no randomized controlled trial has demonstrated an effective means of weight loss that results in improved pregnancy outcomes for both mother and baby. This study intends to determine if substantial pre-conception weight loss, achieved using a VLED, improves pregnancy outcomes for mother and baby when compared with standard care. This research will potentially change clinical care of an obese woman planning pregnancy.

**Trial registration:**

ANZCTR, 12,614,001,160,628. Registered on 5 November 2014.

**Electronic supplementary material:**

The online version of this article (10.1186/s13063-018-2615-6) contains supplementary material, which is available to authorized users.

## Background

Obesity is present in one in three women of child-bearing age [1]. Currently, guidelines for the management of obesity in women planning pregnancy are based on consensus view and lack supporting evidence. It is critically important that we have evidence-based weight loss tools that reduce the risk of obesity-related pregnancy outcomes for both mother and fetus. This study aims to determine if substantial weight loss achieved using a modified very low energy diet (VLED) program fulfils this clinical need.

The impact of maternal obesity on pregnancy outcomes have been well described. Maternal risks include gestational diabetes, gestational hypertension and pre-eclampsia, medically indicated induction of labor, instrumental delivery, and primary Cesarean section [1]. Fetal risks include large-for-gestational age (LGA), pre-term delivery, hyperbilirubinemia, hypoglycemia, and need for admission to the special care nursery or intensive care unit [1, 2]. Maternal obesity also increases the risk of congenital anomalies [3, 4] and perinatal death [5, 6].

Pre-gravid maternal obesity [7], maternal gestational diabetes (GDM) [7], and being born LGA [8] are the most significant risk factors for obesity in childhood. Obesity in childhood is the strongest predictor of obesity in adulthood [9]. Emerging evidence suggests the metabolic status of the mother may “program” the offspring’s long-term risk of metabolic disease [10–12].

To date, studies aimed at reducing obesity-related adverse pregnancy outcomes have focused on limiting gestational weight gain. More than 50 such interventional trials have been conducted but these studies have, at best, been only very modestly effective in reducing adverse pregnancy outcomes. The LIMIT study was a large, well-designed study of lifestyle modification during pregnancy. The study demonstrated no risk reduction for delivering a baby ≥ 90th centile for gestational age and no improvement in maternal pregnancy or birth outcomes. There was also no statistically significant difference in gestational weight gain between the control and intervention groups [13]. Similarly, the UPBEAT trial showed that a program of diet and exercise had no impact on the risk of gestational diabetes and LGA offspring in obese pregnant women [14]. These important studies inform us that alternative approaches to the management of obesity in women planning pregnancy are needed.

A review of five national guidelines from Canada, Ireland, the United Kingdom, Australia/ New Zealand, and the United States of America showed that there are significant differences in the management of obese women planning pregnancy between countries [15]. All guidelines included in the review recommend weight loss before pregnancy using lifestyle modification such as diet and exercise. These recommendations are made on the basis of consensus opinion. The United Kingdom guidelines offer specific advice on how to monitor weight and change health behavior, including the recommendation to reach a body mass index (BMI) of 18.5–24.9 kg/m^2^ before conception [15]. However, for a woman of average height (165 cm) and a BMI of 40 kg/m^2^, a 42-kg weight loss would be required to reach BMI 24.9 kg/m^2^. Lifestyle modification in the Counterweight Programme (*n* = 642) demonstrated a mean weight loss of 3.0 kg after 12 months [16]. Similarly, a lifestyle program in obese infertile women (*n* = 236) demonstrated a mean weight loss of 4.4 ± 5.8 kg over six months [17]. While modest weight loss has metabolic benefits for the woman [18–20], there is no evidence that it reduces the risk of obesity-related pregnancy complications.

Numerous retrospective studies of bariatric surgery before conception have demonstrated a reduction in adverse pregnancy outcomes for the mother [21–25]. The largest study (*n* = 627) demonstrated that bariatric surgery (mean weight loss from surgery to early pregnancy = 37 kg [BMI 43.7 kg/m^2^ to 30.3 kg/m^2^]) was associated with a reduction in maternal gestational diabetes and LGA infants. However, risk of small-for-gestational-age (SGA) infants and possibly perinatal mortality was increased [26]. In this study, the median interval from surgery to conception was 12 months, suggesting that even after conception, rapid surgery-induced weight loss was occurring. Catalano has shown that those women who gain inadequate weight during pregnancy (< 5 kg) have a significantly increased risk of SGA infants [27]. SGA infants are at similar long-term risk of metabolic disease to LGA infants [27].

There is limited experience with the use of medications such as sibutramine, phentermine, and orlistat as an adjunct to lifestyle modification in the pre-pregnancy setting [28, 29]. In these studies, slightly more weight loss was achieved when the medication was used. However, live birth rate was not substantially improved. There are no published studies examining the impact of GLP-1 analogs in the pre-pregnancy setting. However, case studies of inadvertent use of GLP-1 analogs in the pre-pregnancy and early pregnancy setting do exist and no adverse outcomes are reported [30]. This is an area of future potential research [31, 32].

VLEDs result in 10–15% weight loss over 12 weeks in an adult population [33]. Such programs may be particularly suitable for pre-conception weight loss as they promote rapid weight loss which assists program engagement [34], induce sufficient weight loss to improve fertility [35, 36], ensure adequate intake of protein which is associated with favorable pregnancy outcomes if pregnancy occurs [37], and can be stopped before conception, ensuring gestational weight gain is not affected. Some have argued that VLEDs should not be used in women planning pregnancy due to the potential of exposing the developing fetus to ketosis. This concern has been based on animal studies, which show potential harm to the pup when exposed to high-level ketosis for the entire period of gestation and lactation [38]. Women on VLED programs are advised to use contraception. Hence, the early developing fetus would only be exposed to ketosis if unintended pregnancy occurred. Reassuringly, the human fetus is regularly exposed to brief periods of ketosis in the context of hyperemesis gravidarum [39], type 1 diabetes [40], and normal pregnancy [41]. In all such cases, there is no evidence of harm to the fetus. Multiple small studies of VLED pre-pregnancy support this concept [42, 43].

The primary metabolic abnormalities associated with obesity are insulin resistance and hyperinsulinemia [44]. Insulin resistance not only affects glucose metabolism; increased free fatty acids and triglycerides are hallmarks of insulin resistance. The obese woman begins pregnancy with greater insulin resistance than her normal weight counterpart. There is a further 50–60% increase in insulin resistance due to the pregnancy itself [45]. This progressively increases as the pregnancy progresses [46]. If the beta cell cannot compensate for the increase in insulin resistance, glucose levels rise. The developing fetoplacental unit is exposed to these metabolic changes [47].

The seminal Hyperglycaemia and Adverse Pregnancy Outcome (HAPO) study (*n* = 23,316) demonstrated that even within the “normoglycemic” range, small increases in maternal glucose are associated with adverse pregnancy outcomes [48]. Similarly, a small decrease in maternal glucose is associated with a lower risk of adverse pregnancy outcomes. For example, a reduction in maternal fasting glucose from glucose category 5 (5.0–5.2 mmol/L) to glucose category 3 (4.5–4.7 mmol/L) (a 10% reduction in fasting glucose) will result in a reduction in the rate of neonates born LGA from 16.5% to 10.1%, cord blood C-peptide from 17.7% to 8.2%, and the rate of primary Cesarean section from 23.7% to 18.5% [48].

The HAPO data were re-analyzed to investigate the impact of BMI on adverse pregnancy outcomes [49]. This study demonstrated that both maternal hyperglycemia and obesity at 24–32 weeks’ gestation are independently associated with adverse pregnancy outcomes. However, the combination has a greater impact than either one alone. The same association exists if pre-pregnancy BMI (based on patient recall) is considered. Weight loss improves glycemic control [19, 20]. Pre-pregnancy weight reduction has the potential to improve pregnancy outcomes, directly through weight loss and indirectly through improved glycemic control.

Regardless of the method of weight loss, the counter-regulatory responses to weight loss will result in a tendency to weight regain [50]. While any weight regain is not ideal, multiple studies suggest that weight maintenance for 12 months is possible [51, 52]. Importantly, the rate of weight regain is not altered by the rate of weight loss [34]. This would allow a window for conception at a time of lower weight.

In considering weight loss in an obese woman before pregnancy, we must identify a weight loss target that both increases fertility and decreases the risks of adverse pregnancy outcomes due to obesity. This study aims to address this need by exploring the impact of substantial pre-conception weight loss in obese woman on maternal and fetal pregnancy outcomes.

### Aim

Primary aim: To determine if non-surgical substantial pre-conception weight loss (10–15% body weight) in obese (BMI ≥ 30 kg/m^2^) women causes a ≥ 10% reduction in fasting glucose at 26–28 weeks’ gestation when compared with modest pre-conception weight loss (≤ 3% body weight loss).

Secondary aims:

The secondary aims of the study are to determine if, in obese women (BMI ≥ 30 kg/m^2^), non-surgical substantial pre-conception weight loss (10–15% body weight) when compared with modest pre-conception weight loss (≤ 3% body weight), results in:

1. A reduction in the composite end point of maternal gestational diabetes (IADPSG definition), LGA, intrauterine growth restriction (IUGR), gestational hypertension/pre-eclampsia, delivery before 37 weeks’ gestation, primary Cesarean section, shoulder dystocia/birth injury, neonatal hypoglycemia, neonatal hyperbilirubinemia, neonatal special care nursery, or intensive care admission.
2. A reduction in the rate of maternal gestational diabetes (IADPSG definition).
3. A reduction in the rate of LGA infants.
4. A reduction in the rate of IUGR.
5. A reduction in the rate of gestational hypertension/pre-eclampsia.
6. A reduction in the rate of delivery before 37 weeks’ gestation.
7. A reduction in the rate of primary Cesarean section.
8. A reduction in the rate of birth injury/shoulder dystocia.
9. A reduction in the rate of neonatal hypoglycemia.
10. A reduction in the rate of neonatal hyperbilirubinemia.
11. A reduction in that rate of neonatal special care nursery or intensive care admission.
12. A reduction in maternal and neonatal length of stay.
13. A decrease in the time to conception.
14. An increase in live birth rate.
15. No difference in maternal gestational weight gain.

## Methods/design

A two-arm, parallel group, randomized control trial will be conducted in the Metabolic Disorders Centre, University of Melbourne. Obese (BMI 30–55 kg/m^2^) women planning pregnancy in the next 6–12 months will be recruited using social media advertisements (Facebook®) and via referral from a Weight Control Clinic at a tertiary hospital.

Subjects who identify themselves as potential trial candidates will click on the Facebook® advertisement and will be directed to a secure study website. An online ‘Registration of Interest’ form will be completed. This will be received by the study co-ordinator and will trigger a phone screening to ensure that the subject is (a) planning pregnancy in the next 6–12 months (b) lives in reasonable proximity to study sites (c) meets the BMI criteria. Potential trial participants will be sent a participant information and consent form. If they wish to proceed, they will contact the study co-ordinator and a screening appointment will be booked.

At the screening visit, patients will be reviewed by a medical doctor. After signing the consent form, medical history and current medications will be recorded. A medical summary will also be sought from the local doctor. A physical examination will be performed including height, weight, waist circumference and blood pressure. Patients unsuitable for the study will be referred to a weight management clinic in a tertiary hospital.

Inclusion criteria are women who meet the following:

- BMI 30–55 kg/m^2^
- 18–38 years of age
- Planning pregnancy in the next 6–12 months
- Willing to undergo a weight loss program and to avoid pregnancy during this program (16 weeks).
- Living in Victoria, Australia.

Exclusion criteria are:

- BMI < 30 and > 55 kg/m^2^
- Age < 18 and > 38 years old
- Current pregnancy/lactation
- Significant medical or psychiatric illness that would preclude the use of a VLED for 12 weeks
- Current use of drugs/complementary medicines known to impact weight
- Diabetes (Type 1 Diabetes or Type 2 Diabetes)
- Diabetes therapies (except Metformin). Metformin use in the context of Polycystic Ovarian Syndrome (PCOS) is not an exclusion. However, participants will be asked to with-hold the dose on the day before all study blood tests. Participants are not permitted to up-titrate the dose of Metformin for the duration of the study. The dose may be ceased if medically indicated.
- Discretion of the investigator

Withdrawal criteria are:

- The participant wishes to discontinue
- The participant is unwilling or unable to comply with the study protocol.
- The participant misses more than one trial visit during the weight loss phase of the study.
- The participant is pregnant before the completion of the weight maintenance phase. In this case, the pregnancy will be followed but data will not be included in study results.
- The participant is withdrawn at the discretion of the investigator for a medical, psychological or social reason.

### Randomization

Women will be randomized to the two treatment arms (Group A and Group B) using randomly permuted blocks of size 2, 4 or 6 (also randomly chosen) within 6 strata accounting for BMI (30–34.9; and 35–50 kg/m^2^), age (18–29 and 30–38 year-old), and parity (0 or 1+).

#### Group A: Moderate Weight Loss (MWL)

Nutritional advice will be delivered by a qualified dietician in accordance with the ‘Australian Guide to Healthy Eating’. Daily energy expenditure will be calculated based on Basal Metabolic Rate and Activity Level (Harris-Benedict formula). A hypocaloric diet (500 cal deficit from the daily energy expenditure) will be recommended to achieve a weight loss of 0.5 kg per week. A food diary and an ‘Allan Borushek’s Calorie, Fat and Carbohydrate Counter’ book (Hinkler books, 2014) will be provided to participants as tools to assist weight loss. Over 12 weeks, ≤3% of body weight loss is expected. This is current best practice.

#### Group B: Substantial Weight Loss (SWL)

Women will be instructed to replace two meals per day with a commercially available very low energy dietary formulation, Optifast **®** (Nestle Nutrition, Australia). The third meal of the day must consist of 150 g of protein (meat, chicken, fish, tofu, eggs), 2 cups of low-starch vegetables and salad dressed with 2 tablespoons of oil (to stimulate the gallbladder and prevent gallstones). Nutritional advice will be delivered by a qualified dietician. The group will receive ‘Allan Borushek’s Calorie, Fat and Carbohydrate Counter’ book (Hinkler books, 2014) but will be advised that the information in this book will only be relevant after completion of the intervention phase. Total daily calorie intake is approximately 800 cal. Over 12 weeks, 10–15% total body weight loss is expected [33] (Table 1).

**Table 1:** Sample constituents of the VLED product

OPTIFAST® VLCD Shake Vanilla 53 g			
Servings per pack: 12 Serving Size: 53 g (Powder)	Average quantity per serving	Average quantity per 100 g	Ave quantity per 100 mL (made up with 200 mL water)
Energy	840 kJ	1580 kJ	354 kJ
	201 Cal	379 Cal	85 Cal
Protein	20 g	37.7 g	8.4 g
Fat-total	4.5 g	8.5 g	1.9 g
- Saturated	0.9 g	1.7 g	0.4 g
- Linolenic acid	1.2 mg	2.2 mg	0.5 mg
- α-Linolenic acid	196 mg	370 mg	83 mg
Carbohydrate	18.2 g	34.4 g	7.7 g
- Sugars	10.1 g	19 g	4.3 g
- Lactose	9.5 g	18 g	4.0 g
Dietary fiber	3.6 g	6.8 g	1.5 g
Sodium	215 mg	410 mg	92 mg
Vitamin A	345 μgRE	650 μgRE	146μgRE
Thiamin (B1)	0.58 mg	1.10 mg	0.2 mg
Riboflavin (B2)	0.74 mg	1.40 mg	0.3 mg
Niacin	8.0 mgNE	15 mgNE	3.4mgNE
Pantothenic acid	2.7 mg	5 mg	1.1 mg
Vitamin B6	1.0 mg	1.9 mg	0.4 mg
Biotin	10.6 μg	20 μg	4.5 μg
Folic acid	110 μg	210 μg	47 μg
Vitamin B12	1.1 μg	2 μg	0.4 μg
Vitamin C	40 mg	76 mg	17 mg
Vitamin D	3.7 μg	7 μg	1.6 μg
Vitamin E	7.4 mgTE	14 mgTE	3.1 mgTE
Vitamin K	31.8 μg	60 μg	13.4 μg
Calcium	420 mg	800 mg	180 mg
Chromium	13 μg	25 μg	5.6 μg
Copper	1.1 mg	2 mg	0.4 mg
Fluoride	340 μg	650 μg	146 μg
Iodine	98 μg	185 μg	42 μg
Iron	8.0 mg	15 mg	3.4 mg
Magnesium	160 mg	300 mg	67 mg
Manganese	0.8 mg	1.5 mg	0.3 mg
Molybdenum	18.6 μg	35 μg	7.8 μg
Phosphorus	360 mg	680 mg	150 mg
Selenium	40 μg	75 μg	16.8 μg
Zinc	4.2 mg	8 mg	1.8 mg
Potassium	955 mg	1800 mg	405 mg
Chloride	280 mg	530 mg	120 mg
Gluten	Nil detected	Nil detected	Nil detected

### Intervention

The trial will involve three phases.

#### Phase 1: Weight loss (Weeks 0–12)

##### Weeks 0 & 12 (all participants)

On arrival to the clinic, a fasting blood sample (20 ml) will be taken for measurement of plasma glucose, insulin, lipid profile (total cholesterol, LDL, HDL, triglycerides, non-esterified fatty acids (NEFA)), leptin and C-reactive protein (CRP)). The specified period of fasting is 8–12 h with blood samples drawn between 08:00 and 10:00 am. A urine and serum pregnancy test will be performed. Urinalysis will be performed for urinary ketones. Maternal anthropometry (weight, waist and hip circumference, skin fold thickness measurement) and blood pressure will be measured (see Data and Biosample collection 1.1 and 1.2).

Participants will be asked to avoid pregnancy using a medically proven form of contraception. Adequate methods of contraception include barrier contraceptives, hormonal contraceptives (oral, implanted, injectable) or mechanical products (intra-uterine device). The patient’s understanding of this will be documented. Standard pre-pregnancy medical advice for obese women planning pregnancy will be discussed. This includes the use of a multi-vitamin containing iodine 150μg (one tablet PO daily) and high dose folate (5 mg PO daily) [53].

The medical doctor will discuss the national guidelines for exercise with the participant using the NHMRC brochure titled “Australia’s Physical Activity and Sedentary Behaviour Guidelines for Adults”. Participants will be provided with a pedometer (Yamax 200S) and will be asked to wear the pedometer for 7 consecutive days (Week 2 of the weight loss phase). An average step count will be recorded. Exercise does not play a significant role in short-term weight loss but we wish to demonstrate that differences in weight loss between the groups are not due to exercise [54, 55].

Participants will be provided with a survey to take home and complete before the next study visit. This survey is based on the previously published Health in Pre-conception, Pregnancy and Post-birth (HIPPP) Study [56]. It is intended to assess areas including depression, anxiety, body image and activity level, which may impact health during pregnancy. This is expected to take around 1 h to complete. The decision to participate or not participate in this aspect of the study will not impact involvement in other aspects of the study.

##### Weeks 2,4,6,8,10

Maternal anthropometry and blood pressure will be measured (see Data and Biosample collection 1.1 and 1.2). A brief medical history will be taken and medication will be adjusted as required. A urine test will be collected. A urine pregnancy test and urinalysis for urinary ketones will be performed. The dietician will provide ongoing dietary counselling.

#### Phase 2: Weight maintenance (Weeks 13–16)

Both study arms will be asked to adopt a healthy balanced diet based on the “Australian Guide to Healthy Eating” for a period of 4 weeks and will be asked to aim for weight maintenance. At week 16, maternal anthropometry and blood pressure will be measured (see Data and Biosample collection 1.1 and 1.2). If greater than 3 kg of weight regain has occurred, participants will see the dietician for further dietary counselling. At the end of this phase, women will be advised contraception can be ceased and they may try to conceive.

#### Phase 3a: Pre-pregnancy (Week 17–60)

Women from both groups will be seen every 3 months whilst attempting to conceive. Maternal anthropometry and blood pressure will be recorded (see Data and Biosample collection 1.1 and 1.2). Any medical issues and changes in medication will be documented. A urine pregnancy test will be performed. Women will see the dietician to assist weight maintenance. If pregnancy has not occurred within 12 months of Phase 1 of the trial, involvement in the trial will cease. If appropriate, the women will be referred on for fertility care.

#### Phase 3b: Pregnancy (Conception-3 days post delivery)

Women will contact the study coordinator when a home pregnancy test is positive. There is no limitation to how pregnancy is achieved (spontaneous pregnancy, clomiphene induction, artificial reproductive technology). Method of conception and approximate estimated date of conception will be recorded. It is accepted that date of conception may initially be difficult to determine. Subsequent study booking will be based on a derived date when late menstrual period dates, BHCG titre and/or ultrasound results are known. This is what would occur in standard clinical practice. Likely obstetric care provider will be recorded. Medical records including the results of the standard 75 g Oral Glucose Tolerance Test (26–28 weeks’ gestation), all ultrasonography reports and the Victorian Maternity Record will be obtained in all women.

If women elect to attend The Mercy Hospital and Royal Women’s Hospital for obstetric care, they will be asked to agree to an additional four study visits throughout pregnancy.

- Visit 3.1 (12 weeks pregnant): Maternal anthropometry and blood pressure will be measured (see Data and Biosample collection 1.1 and 1.2). A fasting maternal blood sample (20 ml) will be taken for measurement of plasma glucose, insulin, lipid profile (total cholesterol, LDL, HDL, triglycerides, non-esterified fatty acids (NEFA), leptin and C reactive protein (CRP).
- Visit 3.2 (26–28 weeks pregnant): Maternal weight, skin fold thickness measurements and blood pressure will be taken. Maternal bloods will be repeated as for Visit 3.1. A kit for maternal and cord blood collection (containing tubes and instructions) will be given to participants. This will be packed into the baby bag of subjects. This method of cord blood collection has been used successfully in the past.
- Visit 3.3 (Delivery): After the 3rd stage of labour, cord blood (20 ml) will be taken from the umbilical vein using direct puncture of the vessel with needle and syringe. Maternal blood will be taken (see Data and Biosample collection 1.1 and 1.2).
- Visit 3.4 (24-72 h post-delivery): Fetal anthropometry (see Data and Biosample collection 1.3) will be performed by one of two trained persons based on the protocol used in the HAPO study [57]. Maternal anthropometry and blood pressure will be performed (see Data and Biosample collection 1.1 and 1.2). Gestational weight gain will be calculated (to the nearest 0.1 kg).

**Fig. 1:**
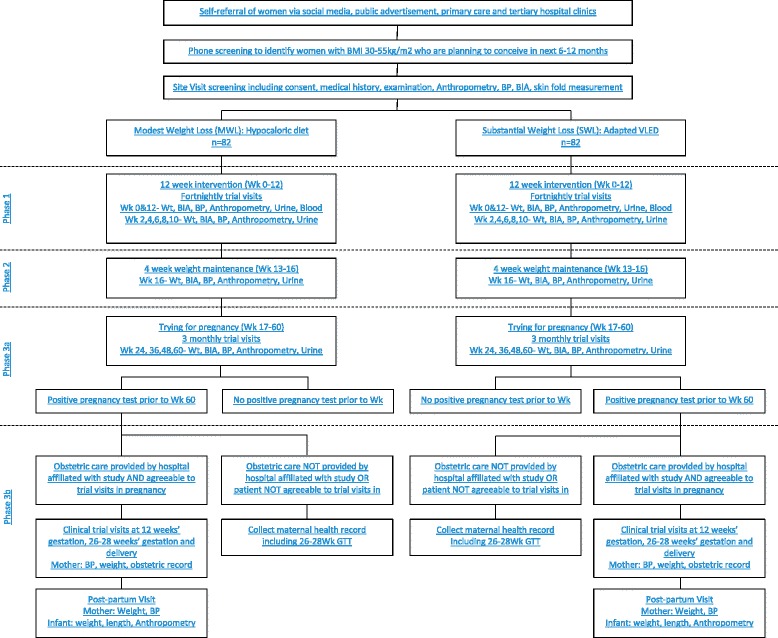
*Flow chart* of study protocol

All standard pregnancy care, including the decision to proceed with an early 75 g Oral Glucose Tolerance Test (OGTT), will occur at the discretion of the treating maternity team. If gestational diabetes is diagnosed at any gestation, treatment should proceed per usual local guidelines. The diagnosis of gestational diabetes will be documented in the participant file and all medical therapy used to manage gestational diabetes will be recorded. Given that these women will not have an OGTT at 26–28 weeks’ gestation, glucose samples from these participants cannot be used in the analysis of the primary outcome. Study samples should otherwise be collected per the study protocol (Fig. 1).

### Outcome measures

The results of the standard 75 g oral glucose tolerance test (OGTT) performed at 26–28 weeks’ gestation will be collected in all participants (except those who had an early OGTT which was positive; these subjects will not have a OGTT at 26–28 weeks’ gestation and therefore these subjects are excluded from the primary outcome). The fasting maternal plasma glucose from the 75 g Oral Glucose Tolerance Test performed at 26–28 weeks’ gestation will be used for analysis of the primary outcome. This test is part of standard maternity care and the sample could be taken in any one of the pathology collection centres across Victoria. Storage and processing of samples will occur according to the standard protocol of the pathology company. While it is acknowledged that samples may be processed in different laboratories using different techniques (hexokinase method or glucose oxidase method), it is anticipated that using this sample as the primary outcome will maximise the number of samples available for analysis. If samples are taken, stored and processed according to the standard protocol of the pathology company, the inter-assay variation is likely to be extremely low [58].

All women who agree to take part in the study throughout pregnancy will also have fasting glucose samples taken at their scheduled 26–28 weeks’ gestation study visit. This glucose sample will not be taken on the same day as the 75 g OGTT but will be taken during the same 26–28 week gestation window. This sample will be taken after an 8–12 h fast and between 08:00 and 10:00 am. Samples will be stored and batch tested as per Methodology 1.4. Analysis of these glucose samples will occur by the hexokinase method. These results will be compared to the fasting maternal plasma glucose samples from the 75 g Oral Glucose Tolerance Test. These samples will be used to validate the glucose results that comprise the primary outcome.

After delivery, the Victorian Maternity record will be obtained in all women who become pregnant. This will provide data on secondary outcomes including large-for gestational age, pre-eclampsia, small for gestational age (SGA), delivery before 37 weeks’ gestation, caesarean section, shoulder dystocia/birth injury, neonatal hypoglycemia, neonatal hyperbilirubinemia, and special care nursery or neonatal intensive care admission.

### Data and biosample collection

Study data will be collected by trained research staff. All blood samples will be taken from the right or left cubital fossa, after an 8–12 h fast, between 08:00–10:00 am. Dietary advice will be provided by appropriately trained dieticians. Personal information will be maintained separately from data, and is accessible only by study co-ordinators and principal investigators.

#### 1.1 Maternal anthropometry

Body weight will be measured (to the nearest 0.1 kg) using calibrated digital scales. Waist and hip circumferences will be measured (to the nearest 0.1 cm) using a standard tape measure according to the WHO guidelines (at the end of a normal expiration, at the midpoint between the lower margin of the last palpable rib and the top of the iliac crest, and around the widest portion of the buttocks, as the average of two measurements). Skin fold thickness measurement will occur according to the protocol published by Kannieappan and will involve triceps, biceps, subscapular skinfold thickness measurements plus arm circumference [59]. Measurements will be taken fortnightly during the weight loss phase (weeks 0–12), at week 16 and then 3 monthly for 12 months or until conception occurs. Post-conception measurements will occur at 12 and 26 weeks’ gestation and within 72 h of delivery.

#### 1.2 Maternal blood pressure

Blood pressure will be measured from the left arm, with an automated sphygmomanometer after participants have been seated for 5 min. All measurements will be taken with a large cuff.

#### 1.3 Fetal anthropometry

Weight will be measured (within 24 h of delivery) on digital calibrated scales. Length will be measured using a measuring board, and head circumference measured using a tape measure. Harpenden calipers will be used to measure skinfold thickness. in the mid-axillary line, triceps fold and subscapular fold. All measurements will be taken on the left side and repeated until a consistent, stable reading is obtained. Neonatal body fat estimation will be performed using the formula derived by Catalano [60]. There is no significant difference between this anthropometric estimate of body fat and total body electric conductivity.

#### 1.4 Laboratory assays

**Fig. 2:**
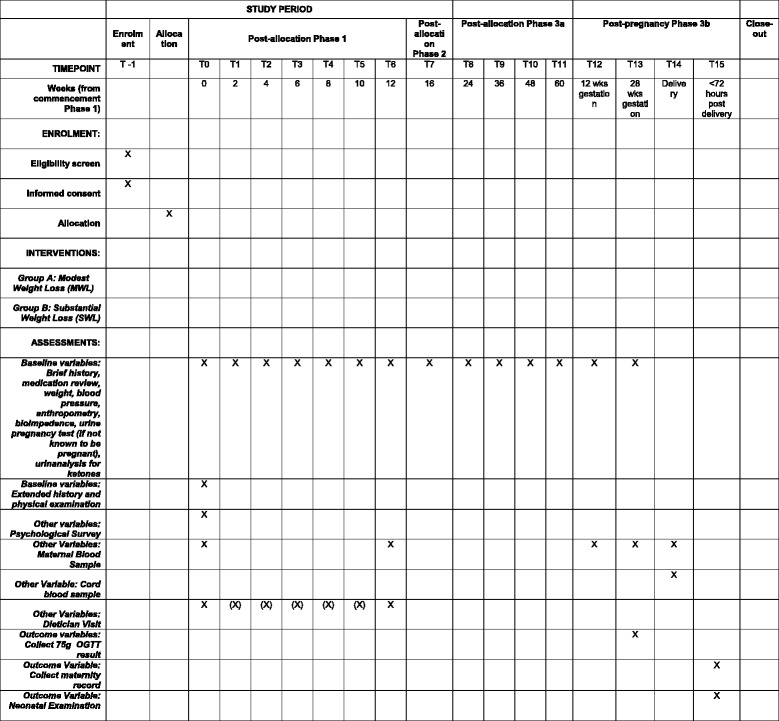
Example of template of recommended content for the schedule of enrolment, interventions, and assessments. X denotes tasks which must be completed in all study participants. (X) denotes tasks that are recommended but are not required

At each visit requiring a blood sample, 3 serum tubes, 1 lithium heparin tube and 1 fluoride EDTA tube with be taken. Samples will be mixed by inversion and centrifuged at 4 degrees Celsius. Plasma from the three serum tubes will be aliquoted into 5 microtubes for insulin/c-peptide, CRP/lipids and leptin with two spare samples. Plasma from the lithium heparin tube will aliquoted into 2 microtubes for free fatty acids. Plasma from the fluoride ETDA with be aliquoted into two microtubes for glucose. Plasma samples will be batched for analysis to minimise inter-assay error. Sample will be stored at − 80 degrees Celsius before analysis. Spare tubes will only be used in the event of a processing error. Our laboratory (Austin Pathology, Melbourne, Australia) has experience in measuring glucose, insulin, lipids, C-peptide, C-reactive protein (CRP), free fatty acids and leptin. The study database allows management of the samples. All analysis will be carried out on anonymised samples (Fig. 2).

### Study management and governance

This two-arm, parallel group, randomized control trial is led by investigators based at the University of Melbourne. This is an academic institution comprising investigators from Melbourne Health, Austin Health, Royal Women’s Hospital, and Mercy Health. All involved institutions are based in Melbourne, Australia. The principal investigators will be responsible for all decisions in regard to management and delivery of the study. Data collected will be re-identifiable (coded) so that it remains confidential but could be identifiable if critical results are noted. All study information is treated as confidential and is securely stored. Data monitoring, protocol modifications, and reporting of serious adverse events will occur according to the specification of research ethics committees. All principal investigators will have access to the interim dataset and any decision to terminate the trial will be made by consensus. Principal investigators will be responsible for communication of the final results study results via publication in peer-reviewed journals.

### Statistical analysis/Power calculation

Most studies investigating the impact of maternal weight on pregnancy outcome have used the incidence of LGA neonates as the primary outcome. If this study used the same outcome, > 2000 women would be required to have sufficient offspring to achieve adequate power. This is clearly not practical. The HAPO data demonstrated a strong and continuous association between maternal fasting glucose at 24–32 weeks and pregnancy outcomes, including LGA neonates. Therefore, change in maternal fasting glucose at 26–28 weeks’ gestation can be used as a proxy for the incidence of LGA neonates.

In our pilot study, Phase 1 of the study demonstrated substantial weight loss (SWL) resulted in a reduction in maternal fasting glucose of approximately 10% (actual 9.12%, SE = 1.83, *n* = 24) compared to the modest weight loss (MWL) group (1.24%, SE = 1.40, *n* = 14). This is similar to previous studies by Sumithran [50] and Purcell [34]. In phase 3 of the pilot study (*n* = 10, MWL 3, SWL 7), glucose reductions were maintained at both 12 weeks’ gestation and 26–28 weeks’ gestation in both groups. Allowing for at least a moderate effect size of 0.6 for difference in percentage decrease in glucose at 26–28 weeks’ gestation (i.e. difference in means = 6, SD = 10; or slightly larger difference in means and larger SD) we require 45 women in each group to achieve 80% power.

We anticipate 45% of participants will not achieve a live birth within the study time-frame. This would include 20% drop-out during the weight loss phase. The anticipated drop-out rate is based on pilot data, on previous studies conducted by Purcell [34] and Rothberg [61], and by the lifestyle interventional study conducted by Mutsaerts [17]. We anticipate a further 25% of participants will fail to become pregnant within 12 months or will experience an early pregnancy loss at < 20 weeks’ gestation. This is based on the data from Kort et al. who found women with a mean BMI of 36 kg/m^2^ had a conception rate of 88% or 54% when losing ≥ 10% weight or < 10% weight, respectively [28]. We therefore require approximately 164 individuals.

The primary outcome will only consider those women who conceive and have a 75 g Oral Glucose Tolerance Test at 26–28 weeks’ gestation. Where appropriate, secondary outcomes will be analyzed both considering only those women conceive and also by intention to treat.

## Discussion

Obesity is reaching epidemic proportions among women of reproductive age [1]. The cost of hospitalization during pregnancy for a woman with a BMI > 26 kg/m^2^ is five times greater than for a woman of normal weight (BMI 18–25 kg/m^2^) [55, 62]. However, hospitalization costs explain only a limited proportion of the total financial costs on the healthcare system. As maternal BMI increases, practical difficulties in providing every aspect of obstetric care increase, such as more antenatal visits, more intensive maternal and fetal monitoring, increased induction of labor, and increased risk of operative delivery requiring bariatric trolleys and operating theatre tables [62, 63]. This amounts to huge healthcare expenditure.

The maternal and neonatal risks of obesity in pregnancy are well documented. There is also growing evidence that obesity during pregnancy is a significant driver of obesity in the next generation [64, 65]. The rising rates of obesity in children and young people, and the financial implications of this trend, have been captured in both prominent scientific publications and the lay press over the past five years. Despite this, we still have no safe and effective tool for weight management in obese women planning pregnancy.

Studies aimed at limiting gestational weight gain typically recruit participants at 12–20 weeks of pregnancy. The metabolic state of the mother in early pregnancy (< 12 weeks’ gestation) programs early placental function and plays a critical role in the metabolic status of the mother in late pregnancy [66]. On this basis, the intervention will occur after the critically important early phase of pregnancy. In order to give women sufficient time to achieve the metabolic benefits of weight loss and for the early pregnancy to be exposed to this metabolic environment, it is clear that weight loss must occur before conception.

VLEDs may be a particularly suitable weight loss tool for the pre-conception period given the potential to induce rapid, substantial weight loss before conception while ensuring adequate maternal protein and micronutrient intake. Given a VLED program can be stopped before conception, fetal growth is unlikely to be compromised. The fertility benefit of VLED-induced weight loss has already been demonstrated [29, 35, 42, 67]. However, it is uncertain if VLED-induced weight loss reduces the incidence of obesity-related adverse pregnancy outcomes.

This study aims to determine whether substantial pre-pregnancy weight loss, achieved using a VLED program, can cause a clinically significant reduction in maternal glucose at 26–28 weeks’ gestation when compared with standard care. The HAPO study demonstrated a strong and continuous association between fasting plasma glucose at 24–32 weeks’ gestation and adverse pregnancy outcomes including LGA neonates [48]. It is impractical to use incidence of LGA neonates as a primary outcome in a study such as this, given the large sample size that would be required. Therefore, this study uses the known association between maternal glucose and pregnancy outcomes to inform us about the impact of the intervention. Other impacts of non-surgical substantial pre-conception weight loss will also be explored including time to conception, gestational weight gain, live birth rate, and the incidence of individual adverse pregnancy outcomes including gestational diabetes, LGA neonates, and SGA neonates.

Should this study demonstrate that non-surgical substantial preconception weight loss achieved using a VLED program is safe, effective, and reduces the incidence of obesity-related adverse pregnancy outcomes, this evidence has the potential to change pre-conception guidelines in obese women planning pregnancy. The use of VLED to achieve substantial weight loss in obese women before pregnancy offers the possibility of reducing the healthcare costs associated with antenatal care while improving maternal and fetal outcomes (Additional file 1).

## Trial status

**Fig. 3:**
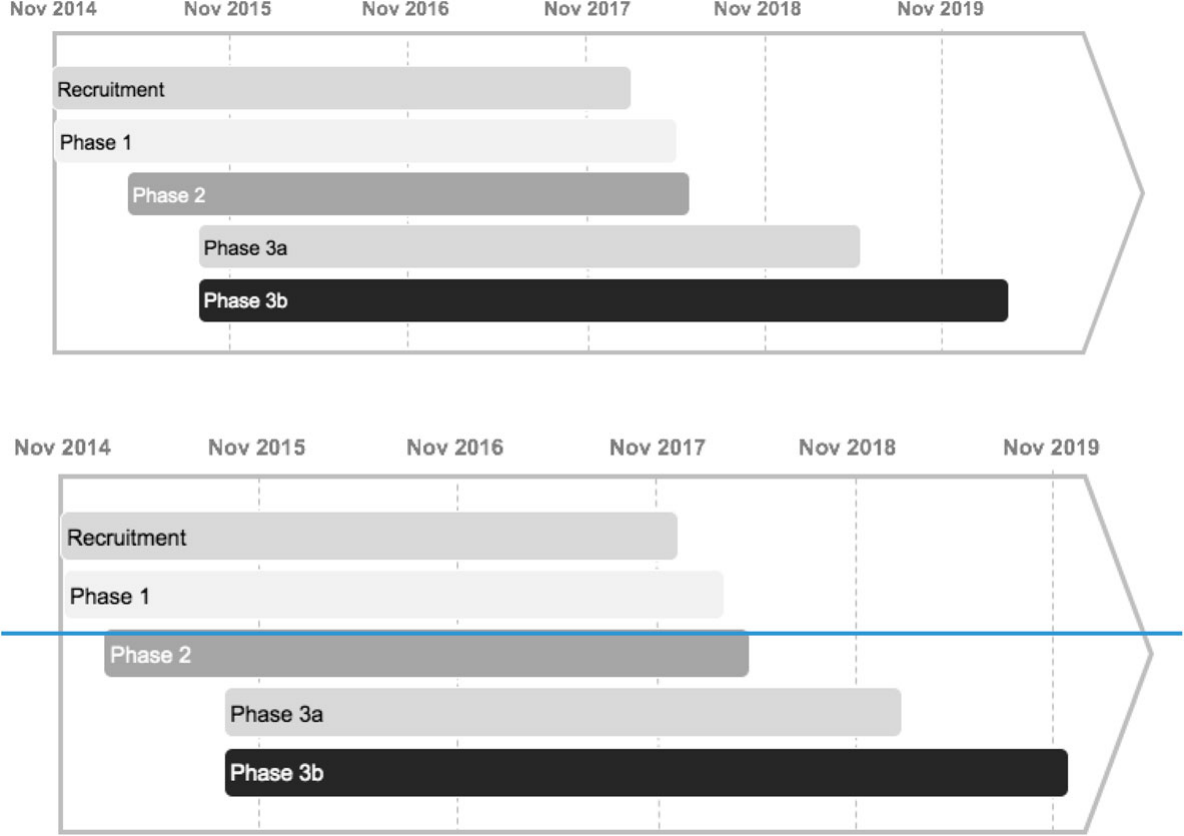
Predicted study time-line

Recruitment began on 5 November 2014. Recruitment will be completed by 15 February 2018. The last participant will complete Phase 1 in May 2018, Phase 2 in June 2018, and Phase 3a in June 2019. Assuming the last participant became pregnant on the last day of Phase 3a, Phase 3b would be completed by March 2020 (See Fig. 3).

## Additional file

Additional file 1: SPIRIT 2013 checklist: Recommended items to address in a clinical trial protocol and related documents. (DOC 122 kb)

## Abbreviations

BIA: Bioelectrical impedance analysis
BP: Blood pressure
GDM: Gestational diabetes
IUGR: Intrauterine growth restriction
LGA: Large for gestational age
SGA: Small for gestational age
VLED: Very low energy diet
